# Ideological dilemmas of policing on the spectrum: Using critical discursive psychology to examine how neurodivergent police manage their identity framing

**DOI:** 10.1177/09579265251376045

**Published:** 2025-10-14

**Authors:** Michelle O’Reilly, Alison Drewett, Samuel J. Tromans

**Affiliations:** 1 University of Leicester and Leicestershire Partnership NHS Trust, UK; 2 Loughborough University and Leicestershire Partnership NHS Trust, UK

**Keywords:** autism, ADHD, discourse, police, disability

## Abstract

In the context of autism and ADHD, tensions exist between medical and social model framings of disability. This article explores neurodivergent police accounts of how aspects of their conditions intersect with their occupational identity and what this means for a disability positioning. Using critical discursive psychology, we illuminate three ideological dilemmas from reflective interviews with 37 autistic police or those with ADHD: (1) tensions between the medical and social identity; (2) tensions between constructing ability and disability; and (3) tensions between remaining invisible and being an advocate. We conclude that a focus on discourse illustrates that organisations need to move away from binary thinking in relation to ability and disability and reconcile some of the tensions navigated by police employees.

## Introduction

There are tensions in how society perceives and treats individuals with disabilities which contributes to inequalities, stemming in part from challenges in attaining and retaining employment (Gov.UK, 2023). Inequalities may be exacerbated for those with psychiatric disabilities, especially where constructs of disability are contentious, like for autism and attention deficit hyperactivity disorder (ADHD); where employment rates are very low (Helgesson et al, 2023; Roux et al., 2013). Understanding employment inequalities for this group is difficult, particularly as there is limited knowledge regarding how challenges may be experienced differently for demanding careers. Indeed, the small, albeit growing literature on high-demand careers in this context does show some important issues raised in areas like medicine (see Doherty et al., 2021), social work (see Guthrie et al., 2025) and policing (see Tromans et al., 2023).

In this paper we focus on policing because this is one site whereby medical model understandings of disability and societal reflections on what is ‘normal’ and ‘deviant’ are prominent. Additionally, research in the context of autistic police and those with ADHD is limited; with most work focusing on how police interact with victims, witnesses or suspects (Crane et al., 2016; King and Murphy, 2014) rather than officers and staff. This is despite estimates suggesting 40% of police employees could be neurodivergent (Broderick-Spencer, 2024) and research showing benefits to policing organisations by having neurodivergent staff (Tromans et al., 2023).

### Background

Medical culture plays a fundamental role in constructing disability. In medicine, underpinned by the medical model of disability, it is assumed that the disability is located within the person, requiring a treatment or cure (see Tan, 2024). Problematically if medicine cannot ‘fix’ the problem, the person becomes viewed as deficient (Gilson and DePoy, 2000). Critical perspectives of disability have, however, claimed that this deficit focus is misleading (Grue, 2011). Social model understandings challenge the power of medicine, alternatively seeing disability as socially constructed (Lester and O’Reilly, 2021a), positing that it is the social environment rather than individual deficit imposing limitations on the person (Oliver, 1981). Arguably, therefore, it is more appropriate to view disability as multidimensional (Altman, 2001), created in and through cultural ideology and discursive positioning (Thomas, 2002). This paradigmatic shift critically questions the role of medicine to empower those with disabilities (Riddle, 2020).

Within discourses of disability, one area courting controversy is that of neurodevelopmental conditions where there are considerable tensions about the role of medicine (Lester et al., 2015; Tan, 2024). Specifically, diagnosis is deterimined by clinical classification. Diagnostic manuals, the 5th edition of the Diagnostic and Statistical Manual of Mental Disorders (DSM-5; American Psychiatric Association (APA), 2013), and the 11th edition of the International Classification of Diseases (ICD-11; World Health Organization (WHO), 2024), clinically define the parameters for diagnostic thresholds for all mental disorders. These manuals define individuals as having a ‘psychiatric disability’ and these manuals include neurodevelopmental conditions. While there are multiple neurodevelopmental conditions, two of the most common are autism, with a global prevalence of approximately 0.8% (see Santomauro et al., 2025), and ADHD, with a global prevalence of approximately 5.3% (see Polanczyk et al., 2007). Autism and ADHD frequently co-occur, with estimates suggesting 40.2% of autistic people have co-occurring ADHD (Rong et al., 2021). Thus, medical classification systems play an important role in determining who is neurodivergent.

Through these classification systems, autism and ADHD are medicalised (Strong, 2014). Medicalisation holds an important place in critical conceptualisations of health and illness by drawing attention to the social causes underpinning the power of medicine (Busfield, 2017), determining what counts as sickness (Nettleton, 2013). Diagnosis becomes the ‘powerful social tool’ occurring between the illness and disease, between the psychologist/psychiatrist and the patient, and provides a cultural expression of what society accepts as ‘normal’ (Jutel, 2009). For autism and ADHD, medical confirmation through diagnosis is the responsibility of psychiatry and psychology. For some this is valuable, as acquiring a diagnosis mobilises resources (Lauchlan and Boyle, 2007) and access to service support (O’Reilly et al., 2020), but creates concerns about the associated disability framing, particularly in a society that favours productivity.

In a neoliberal society, constructing autism and ADHD in terms of deficit and disability has consequences for inequality. In Western economics, it was during the 1980s that a rise in neoliberalism saw social welfare systems dismantled (Evans, 2017), and individuals positioned as responsible (Goodley, 2011). These neoliberal political doctrines created increased income inequality and reduced social cohesion, which impacted interaction with health and healthcare (Runswick-Cole, 2014). Such neoliberalism locates the disability as an individual problem (Ramanthan, 2010), ignoring the social, cultural and ideological context (Nettleton, 2013). Although equality in contemporary society is desired, autistic and ADHD members remain subject to great levels of inequality (Lester and O’Reilly, 2021a). Nonetheless, for autism and ADHD, the social model and related critical arguments are creating change. For example, a modern strengths-based understanding is replacing deficit-thinking, partially due to the political advocacy of the neurodiversity movement, which promotes seeing difference over disability, and challenges the inequalities created by neoliberalism.

Despite progress, economic inequality remains, driven largely by educational and employment disadvantages. Autistic individuals and those with ADHD have greater challenges in gaining and retaining employment (Nicholas et al., 2019; Ohl et al., 2017) because businesses operate through a neurotypical lens (Lorenz et al., 2016). To combat this, employers need to capitalise on the benefits these individuals bring to the workforce (Johnson, 2022) and adopt a strengths-based approach to encourage inclusion (O’Reilly et al., 2025). To some extent, this requires a different way of thinking about disability. Indeed, there are complexities in categorising autistic people and those with ADHD as disabled with some advocates arguing that neurodivergent persons should not be classified as disabled at all, rather as different (Hughes, 2021). This reflects debates regarding the classifications of autism and ADHD in terms of impairment, with differences of opinion expressed (for debates see Lester et al., 2015; and see Tan, 2024). Notably, neurodiversity advocates do recognise that for some individuals there can be a disabling impact of the condition in *some* areas of their lives (Jaarsma and Welin, 2012), which may affect work. Arguably, therefore, there is a distinction played out between disability as a medical experience and being disabled as a social identity which comes with group membership. In other words, if society reconfigures disability as a social category arguably this changes the psychological experiences of the individuals positioned as disabled as it pushes against reductionism of the social reality and reconstructs disability as a complex subjectivity rather than an objective pathology (Dirth and Branscombe, 2018). Dirth and Branscomb noted that social identities are not pre-existing or static in the social world and people with disabilities are viewed as insiders and experts playing important roles in constructing their own identities for collective action.

Regardless of the social model reframing, employers are required to treat autism and ADHD as disabilities within medical categories under the Equality Act 2010. We argue that part of the challenge in promoting employment equality for autistic people and those with ADHD has been in the limited research into specific career choices and employment experiences. Research on neurodivergence and employment has largely clustered employment categories, like skilled/unskilled, or into professional groupings like technical, secretarial, trade and elementary (ONS, 2021). This fails to account for nuanced differences of experience across career and job types and thus it is necessary to undertake research with different occupational groups.

### Focus of the paper

Our focus on autistic police and those with ADHD is centred on the tensions between medical and social model framings of disability, requiring a discursive approach to innovate in this area. We explore how police navigate the boundaries of social and medical model positionings of their condition and their identity. This recognises that viewing medicalisation as ‘good’ or ‘bad’ requires a critical assessment better understood by listening to those who live it.

We ask, ‘how do autistic police officers and staff and those with ADHD navigate the intersections between medical and social model understandings of their neurodivergent identity at work?’

## Method

### Data collection

We worked with the National Police Autism Association (NPAA), an organisation dedicated to supporting police with neurodevelopmental conditions (autism, ADHD, dyslexia, tic disorder etc.). During our first phase we distributed a survey for those who were autistic or had ADHD (see Tromans et al., 2023). We focused on autism and ADHD because they often co-occur together and are common neurodevelopmental diagnoses, as well as to address gaps in the literature regarding employment. From the survey, we recruited 37 participants for interview from across the UK, police officers (*n* = 26) and staff (*n* = 11); 22 identified as autistic and 13 as having ADHD (seven reporting both conditions), and two identified as neurodivergent without being specific. Five participants did not have a formal diagnosis, either because they were on a waiting list or had chosen not to pursue one (three for autism and two for ADHD).

We utilised a reflective interview approach as this provides space for participants to reflect on their experiences (Roulston, 2010). This allowed for a participant-driven conversation. Questions were clustered around their autism or ADHD, career with the police, perceptions of inequality, and organisational support. Interviews were online, usually lasted 1 hour and were audio-recorded and transcribed verbatim.

### Analytic method

We argue that to value participant voices and reflect on tensions, an analytic method is needed that allows for the nuanced, complex, and sometimes contradicting ways in which neurodivergent identities are played out in real world employment. To that end, we utilised critical discursive psychology (CDP) engaging with the practice of ideological dilemmas. CDP is a strand of discourse analysis, combining conversation analysis, discursive psychology and Foucauldian discourse analysis (Wetherell, 1998; Willig, 2013). CDP attends to the ways in which interaction is a form of social action underpinned by wider ‘macro’ aspects such as culture and history (Locke and Budds, 2020). Locke and Budds noted that CDP recognises the socio-cultural context in which talk is produced. CDP is typically underpinned by social constructionism with a focus on language and social interaction (Wetherell, 1998). This is especially useful for the study of mental health, because psychiatric categories are produced and reproduced through language (Harper, 1995). Indeed, autism and ADHD are social constructs and a more critical understanding of how these are navigated in practice goes beyond medical classification (Lester and O’Reilly, 2021a) and benefits from a discursive lens (Lester and O’Reilly, 2021b).

CDP relies on three concepts: interpretive repertoires (embedded social normative scripts people use in talk), subject positions (the mechanism by which people ascribe and are ascribed categories) and ideological dilemmas (the contradictory ways people navigate through talk) (Wetherell, 1998). The concept of ideological dilemmas coined by Billig et al. (1988) is the discursive mechanism through which we organise our analysis. Billig et al. (1988) noted that much discourse is organised around dilemmas as speakers navigate contradictions and these reflect the cultural belief system and social norms of a society. Health and illness are ideological and dilemmatic, and these dilemmas constitute the world of inequalities in the sense that the notion of illness reflects an ideological value, and health beliefs make persons accountable to others (Radley and Billig, 1996). In other words, the prevailing norms of society dictate that people ought to be healthy and productive, contributing to society and when ill, need to take steps to improve with a return to normality being ideological. Thus, the dilemmatic aspects of common-sense are not confined to the social beliefs of a community, but everyday discourse can revolve around sets of oppositions to be navigated (Billig et al., 1988).

To develop our CDP, we initially utilised the approach of unmotivated looking associated with conversation analysis (CA), which is an inductive exploration of the entire data corpus (Schegloff et al., 1992). However, following some noticing of specific issues, like disability and health in the workplace, we viewed the data through a more critical lens, exploring how different dimensions and issues were made relevant by participants in dilemmatic ways. Thus, while the principles associated with CA provided a useful starting point and are aligned with CDP (see Wetherell, 1998), the critical theoretical orientation requires a stronger focus on identifying dilemmas. Thus, in applying CDP, we trawled the data and found that participants described various tensions in relation to health and policing. We therefore paid more specific attention to *how* these tensions were navigated by participants and the ways in which the dilemmas were discussed. Thus, we examine how contrary and sometimes conflicting ideas of health and employment are represented, negotiated and managed in the talk.

### Ethics

Ethical governance was provided by the University of Leicester. All participants provided written informed consent and confirmed on the recording. The NPAA supported recruitment. We refer to participants by number for anonymity.

## Findings

Three ideological dilemmas were noted: (1) tensions between the medical and social identity; (2) tensions between constructing ability and disability; and (3) tensions between remaining invisible and being an advocate. Participants navigated different aspects of themselves and expressed tensions in relation to their beliefs about their autism and/or ADHD. This was in terms of the possible consequences for their job and framed against wider societal, medical and cultural expectations of health and illness and culturally dominant ideas related to policing. They navigated managing their personal and professional identity within those discourses. Thus, participants reproduced dominant medical ideologies while resisting and contesting aspects of them.

### Ideological dilemma one: Tensions between the medical and social identity

Participants oriented to wider societal constructions of neurodivergence in terms of stigma, medicalisation, and the possible inequalities that these labels may create. In so doing, they oriented to the power of medicine in defining expectations of neoliberal productivity and ‘curing’ or treating illness, and cultural ideas about their functioning in the role. The tension grounded in ideology, therefore, was that the individual person reflects a different reality against the wider social norms and beliefs about them. Participants spoke in varied ways that identified their autism or ADHD in medical terms which was often constructed as being in some way associated with deficit and oriented to this being a wider outsider viewpoint. This was undertaken while simultaneously orienting to a social model framing where participants constructed their autism or ADHD as a difference which created challenges for the neurotypical thinking associated with the policing environment. In that sense, therefore, participants discursively positioned the tension between their insider view that neurodivergence is simply a difference, and the ideological outsider views of society reinforcing medical constructions of deficit and disability.

On one hand, some valued the label and medication in managing some of their neurodivergent characteristics (consistent with the medical model), and yet on the other, they proposed that it was the environment in which they worked that created challenges (consistent with the social model). A good example is the way in which participants pitched the neurotypical world in contrast to the neurodivergent one.

But one of the questions was ‘would you want to be cured of your autism?’ and they all said no, because they were like, it’s not the autism that I struggle with, it’s the being autistic in a neurotypical world, that’s the problem. (PS2)

One way in which the dilemmatic aspect of the medical and social model discourses played out was through raising concerns about the insider viewpoint (i.e. the perspective of the individual), against the outsider viewpoint (i.e. the perspectives of their colleagues or wider organisation). In other words, they pitched the tension of their own insider viewpoint in terms of their personal experiences, against the wider outsider societal perspectives that embed medicalised notions of disability in relation to constructs of ‘normality’. Indeed, this was noted in the neurotypical questioning about being *‘cured of your autism’* reflecting the ideology that medical conditions require a return to normalisation. This was strongly resisted by PS2 who reproduced the social model of disability framing to counter such a discourse of being fixed, noting it is the *‘neurotypical world’* that creates challenges not the autism.

This challenge of the insider perspective against the outsider societal view in autism was further captured by PO16. Here PO16 grappled with the dilemma of conceptualising his difference as a ‘*problem’*, which has a deficit overtone. The positioning by others when ascribed was pejorative by suggesting the person has a ‘*problem*’.

It’s just weird because I never knew I had a problem until I was told I’d got a problem. That’s the weirdest thing that I’m trying to cope with at the moment. But I don’t feel, not that I’ve got a problem but I’m just different. (PO16)

Notable in terms of the tensions between the insider experience against the outsider sociocultural construction of autism being a deficit, is PO16’s disclosed ignorance of the proposed idea that he has a ‘*problem’*. Here PO16 suggested that the proposition of him having a problem was *‘weird’* as although his colleagues suggested this was the case, *‘I was told I’d got a problem’*, it was not immediately obvious to him. PO16 notably repositioned his autism as being ‘*just different’*, taking issue with the linguistic framing rather than the conceptualisation of their characteristics more fundamentally. In that sense, he owned being different but denied the problematic framing of that difference, reconstituting a more medical construct ‘problem’ to a social one ‘difference’.

The use of language was a challenge for participants as they navigated the social meanings discursively ascribed to them and their neurodivergent peers. This can be seen in the concerns raised by PO1 who believed that society and police organisations were ‘*disrespectful’* sometimes in categories they employed.

I hate that phrase, high functioning . . . It’s so disrespectful to people who are not maybe as academically able as people at the other end. And also, I may be classed as high functioning if that’s what you call it, but I have days where I don’t feel like I’m functioning very well to be honest, and it really fluctuates depending on what’s going on in your life. (PO1)

PO1 created a contradictory way of considering the notion of ‘*functioning*’, an older phraseology associated with autism. Historically, autistic people were conceptualised as high functioning if they could conform to the norms of society, like being educated, having relationships and holding down a job. In modern parlance, more nuanced conceptualisations are used. For example, individuals may be classified as having low support needs in many domains of life, but medium-high support needs in other areas (Tan, 2024). PO1 considered simultaneous medical and social model understandings of what it means to function, showing that the concept of functioning is typically associated with autism, yet an individual can *‘fluctuate’* depending on circumstances, much like neurotypical persons.

Resisting a problematic framing of autism or ADHD for some meant navigating a dilemma of the dominance of medicine and its reliance on medication. Some participants reiterated the clinical framing of their neurodivergence, noting the role of psychiatry in diagnosis and value of medication. This was especially noted by those with ADHD where medication tends to be more prominent. However, there was tension displayed regarding the role psychiatry plays in determining who does and does not fit into a medical diagnostic category or how to manage the challenges that arise because of it. A good example of this was from PO9 who reported taking stimulant medication for her ADHD and reported recognising its value, describing it as *‘life changing’* in a positive way. In so doing, she reported her insider perspective about the role medication played in managing the characteristics of her ADHD.

I’m on a stimulant now. Which has been life changing. (PO9)

Alternatively, the outsider cultural view problematising neurodivergence was made relevant in the context of taking prescribed medication where there were concerns from the organisation’s perspective about officers taking medication. This was viewed as challenging, as noted by PO13. This tension highlights the larger social struggle at an ideological level faced by individuals between how medication and its effects are conceived within a cultural framing, and the more local specific level of the organisation in terms of its understanding around what is safe and legal in the context of the individual employed with personal experience.

So yeah, occupational health just would not make any decisions because of the meds that I was then on, my ADHD because they’re amphetamines. They were like ‘well we’re not sure if you should be driving on them’. . . . And I was like I’ve passed a driving test, passed my advanced course, been an advanced driver for 18 months or 2 years with no incidents so how have I not shown competence? ‘Oh well okay that’s fine, let’s see how you get on with your meds’. (PO13)

Taking medication in some ways was dilemmatic for PO13, as the medication benefited the ADHD, but meant that she was critically questioned about her competence to do the job. Here PO13 shows that she had been performing well and questioning this was argued to be inappropriate. Notably, most participants did not come to know of their condition until they had been working with the police for some time, and medicating was therefore prescribed during their employment. The dilemma expressed by PO13 was the navigation between locating the medical characteristics within the individual against the wider macro-social environment within which those personal experiences are encountered.

Medicine is a powerful discipline for determining what counts as illness (Nettleton, 2013) and despite the value of medication in managing some characteristics associated with ADHD (or autism), the role of psychiatry was critically considered by some. This was especially the case in terms of conforming to outdated criteria to validate their neurodivergence. For example, PO14 considered the forms she needed to fill in to acquire some reasonable adjustments.

Yeah, and still now because it was set in stone however many years ago by some psychologist or psychiatrist, we still go by all of these different formulas, or the medical practitioners look at that and go ‘well if you don’t tick that box, you’ve not got it’. That’s bollocks. Because it’s really outdated, and it annoys me even now when I have to answer all these forms. (PO14)

Here we see that the police organisation was viewed as aligning with a medical construction of ADHD, as they were reported to utilise forms created by *‘some psychologist or psychiatrist’* and used by police *‘medical practitioners’* for decision-making. The dilemma for PO14 was to have characteristics that were ‘medical’ enough for the form, which was posited as outdated, and yet consider the social reasons for filling in the form which was to have medical validation on paper. Thus, at a meta-level PO14 oriented to the wider ideological debate about disability and difference. She argued that police organisations need to work harder to keep up with the change in social attitudes and recognise the incompatible views that dictate realities, ideologies and beliefs about autism and ADHD.

Relatedly, ways in which policing organisations operate was considered in terms of the social model, as some participants positioned themselves as outsiders to the normative groups within forces. They argued that within policing organisations, neurotypical police were favoured over neurodivergent police as promotion within the force was founded on ‘*personality’* as PO10 argued, but did not define.

I know people in this force . . . who will not promote you because based on your personality, who likes you at that time. That’s wrong. And unfortunately for most neurodivergent staff know that your personality is not going to fit, if you’re not going to change. (PO10)

PO10 argued that neurodivergent people tend to have personalities that fail to conform to the norms favoured by organisations and thus they are positioned on the outside of social favour, especially given notions that personality is seen as a fixed aspect of self. The idea that people can flexibly adapt and ‘change’ to fit into an organisational profile and work in ways that might support a favourable promotion was one argued to cause greater levels of masking. This aligns with neurotypical values that inform the ways in which neurodivergent staff are positioned in other sectors of society. Thus, the medical constructions of autism or ADHD become blended with the operation of policing and police culture.

From this dilemmatic challenge of navigating both medical model and social model constructions of the self, at times participants oriented to an acceptance of and reproduction of the medical perspectives and other times resisted this. Here they described their own experiences of autism and ADHD, positioning themselves as ‘different’, while contrasting that with medicalised ideas of deficit and them having ‘a problem’. This aligns with broader critical challenges regarding what constitutes ‘normal’ and how that permeates a powerful ideology of health and conformity, as well as in terms of who holds responsibility for such validation. The struggle encountered by the participants indicates how medicalisation and pharmaceutical treatment rest on the initial categorisation of the medical deficit with social institutions and employing organisations reifying these constructions. It is via the discursive constructions of neurodivergent individuals grappling with these tensions that we see how these ideological ways of thinking are recreated within policing.

### Ideological dilemma two: Tensions between constructing ability and disability

A strengths-based approach is often polarised against a deficit-understanding of autism and ADHD. As aforementioned, both autism and ADHD are clinically constructed as psychiatric disabilities through the medicalised institutional business of diagnosis, and yet the extent to which individuals ascribe this positioning to themselves, and their conditions is variable (see Lester et al., 2015). The participants’ framing of themselves as disabled and as having strengths in the job was a dilemma reproduced in various ways, from explicit statements to more nuanced presentations of ability and disability.

My autism is a blessing and a curse at the same time in the job. (PO23)

The dilemmatic way in which P023 presented his views about his autism is an interesting one in the context of policing. Utilising emphatic and moralising idioms ‘*blessing*’ and ‘*curse*’ functioned to highlight the dual ends of the spectrum of strength against challenges. Notably, one way in which ideologies of what it means to be disabled was navigated by participants was to note the medical framing of disability while simultaneously critically questioning the extent to which that applied to them.

. . . it’s not necessarily a disability for me, I don’t see it like that. I know it technically is or at least on paper you could call it that way, and there’s all this. . .. . .but no. it doesn’t stop me from doing anything, in fact if anything it’s helped me. (PO3)

Here we see PO3 grappling with the medicalisation of his autism as a disability. PO3 questioned this and instead, he reconstructed his autism as an ability that has *‘helped’* him. By orienting to disability as an impairment, PO3 directly contrasted that by showing that not only does the autism not impair his ability to do the job, the characteristics instead ‘*help’* in that regard. Similarly, PO12 resisted the subject position of ‘disabled’.

And he said [manager] ‘well I’m glad you told me this’ and he sent me this link and went to the disabilities site where you could claim money for, to be able to keep your job, to do your job basically. . . And it was part of the disability. . .. . .And I thought ‘I’m not disabled I think you might be wrong, not me you might be wrong’. And I don’t like it being termed a disability. (PO12)

Navigating the tensions created by the dilemma of ability and disability was positioned by some as a social justice issue as they demonstrated that while they viewed themselves as able, others around them ascribed a disability position. They noted that this was problematic in a hierarchical organisation like policing, as managers attributed disabled identities on disclosure and framed this as a way of mobilising support. Here we see PO12 navigating the way in which his manager positioned him as disabled and in an ostensibly helpful way directed him toward support for those with disabilities. However, this was contrary to PO12’s sense of self with an explicit rejection of disability *‘I’m not disabled’*.

Nonetheless, participants recognised the social model reframing of neurodivergence as an ability was also potentially problematic in the contradictory space of policing whereby medical constructions drove decision-making. They found that in some spaces support was necessary to help with aspects of their autism or ADHD, but that this need did not mitigate the misconceptualisation of disability.

So, when I went to. . .I was like how do I raise this because it didn’t ask. . .it asked me if I had any disabilities when I applied, now I don’t look at it as a disability because actually as a police worker it’s a credit, it’s a really helpful skill to have. (PS6)

Here we see PS6 navigating different ways in which disability and ability are conceptualised and the implications for her own identity in the police. In response to direct questioning from her organisation about disability, the personal perception of PS6 was that there was no disability to disclose. Indeed, disclosing a neurodevelopmental condition to organisations and its members can be risky for some, with fears regarding the possibility of stigma and pejorative attitudes (see O’Reilly et al., 2025). PS6 not only actively denied having ‘*any disabilities’* but argued that the characteristics of her condition were a *‘credit’* to the organisation. Interestingly, distinguished here is the disability and the disabled identity, as PS6 accepted being autistic, which is the clinical condition, but reconstructed this as not being *‘a disability’* thus challenging the way in which the category is applied.

In projecting a strengths-based discourse of their neurodivergence, participants built a case that policing organisations need to work in ways that move away from dichotomising ability and disability, and instead capitalise on their strengths in the role. Indeed, many participants identified areas of policing where they positioned themselves as an asset, and other areas where they noted that characteristics of their condition may impair their ability to do the job well. PO8 provided a clear example whereby an aspect of his ADHD way of thinking was presented as an ideal approach for computer-based problem-solving tasks, but reported that he does have ‘*weaknesses’*.

So, for my strengths are sort of problem solving, and computer stuff, you know. If something needs organising on an excel spreadsheet then I’ll do it. (PO8)

I have weaknesses I’m not going to sugar coat it, but I do have strengths and that’s why I’m lucky to be in the job I am now because I can utilise those. . . (PO8)

By presenting strengths and limitations of ADHD, PO8 navigated the dilemma of ability and disability, adopting a strengths-based discourse *‘my strengths’* while noting the ADHD means ‘*I have weaknesses’*. Indeed, there are characteristics of ADHD (and autism) that can benefit team working as diversity in teams provides a holistic skillset to addressing complex problems, and when those assets are harnessed and utilised effectively, stronger outcomes can be achieved.

For this ideological dilemma of managing tensions between ability and disability, it is notable that at a social level in western society the medical worldview is one that looks from the outside regarding what is safe or ‘normal’ through a lens of the biopsychosocial, and this is reflected through a range of institutions, including police organisations. For the participants, they are navigating how they make sense of their world in terms of what they reported they can or cannot do, or should or should not do. Through this navigation it can be seen that these participants were managing the dilemma of ability and disability in their personal employment situations. Indeed, there was a contrast made between having a medical condition characterising them as disabled against an acceptance of being ‘different’.

### Ideological dilemma three: Tensions between remaining invisible and being an advocate

Not all disabilities are visible, and for autism and ADHD some individuals hide this. Navigating visibility was viewed differently across the participants, with some favouring the invisibility afforded to them to hide their condition from their neurotypical peers, and for others was perceived as more frustrating as they felt they were pushed into situations of advocating their needs and of others. Part of the challenge raised was the misconceptions and attitudes of other police, as they reported that they were sometimes viewed negatively when advocating their need. PO13, for example, reported that her colleagues positioned her in negative ways due to misattributions associated with ADHD.

As I was going out the gate, I can feel that I’m like my hands are shaking, because I can just feel the irritation and the hostility because he was perceiving me again as being argumentative, and all I was trying to do was advocate for myself, my needs. (PO13)

PO13 navigated the dilemma of invisibility and advocacy in noting that the reactions of her colleague were negative due to a misperception of the advocacy. In reporting standing up to ‘*advocate’*, PO13 argued an ‘*irritation’* and ‘*hostility’* from her colleague.

The pejorative ways in which neurodivergent people are reported to be viewed by society was seen as problematic by many and as a reason for maintaining some level of invisibility of the autism or ADHD. This invisibility was for some seen as necessary. For example, PO18 positioned herself as an observer of mistreatment of other neurodivergent police and reported an active effort to stay away from such issues, although seemingly inevitably accepting the fate.

I really struggle with the way that I see people being treated. And although it’s not me at this moment in time, it almost feels like it’s only a matter of time until someone will hate on me again. (PO18)

The use of the term ‘*again’* suggests PO18 has previous experience of ‘*hate’* because of her autism. There was some vagueness in the reporting which implies of poor treatment, without specific examples. In so doing, there is an implied benefit to invisibility, despite being potentially at the cost of support.

Others, however, were more active in advocating for themselves and others. Some highlighted ways in which they challenged the pejorative attitudes and mistreatment. PO7 noted that this challenging of attitudes is important, especially as police officers are dealing with neurodivergent members of the public.

Because it’s not an obvious, because it’s not physically obvious, because it’s my brain, it’s overlooked all the time. All the time. So short of wearing a badge. . .. . .and that is what I find frustrating, I don’t hide it, I actively promote it. I challenge it, if there’s been comments in the main office, ‘oh fucking hell I’ve got to deal with this. . ..’ ‘Well, what’s the job you’re dealing with?’ ‘oh but the victim’s autistic’ and I’ve just gone ‘I’m autistic. . ..’ Like ‘and?’ and they’ve gone ‘oh right ok’. And so, I see it as an educational thing. (PO7)

In navigating the lines of invisibility and advocacy, PO7 promoted the need for change, and proposed that this is only possible through education. By owning their autistic identity, PO7 illustrated how this challenges some thinking by his colleagues in the policing space. In so doing, PO7 suggested the invisibility of autism is itself problematic as it provides a mechanism for ignorance. He argued that because autism is not ‘*obvious’*, the condition gets ‘*overlooked all the time’*. This was felt as ‘*frustrating’* and requiring challenge. Notably, the solution was viewed to be *‘an educational thing’*, with education an important way to reconcile the invisibility advocacy dilemma.

Participants saw education as a mechanism for culture change and change in police values to bring autism and ADHD out of hiding to make it a safer space for people to do so. Notably, PO13 argued that it is the *‘whole culture’* in the police that requires change.

The whole culture and attitudes needs changing, you can’t do that overnight, can you? But I think starting with training would be big thing. (PO13)

Educating police organisations was viewed as crucial for invisible disabilities, as participants noted decisions to disclose relied on improvements in the way police think about autism and ADHD. For example, PS4 argued that police organisations were more responsive and supportive of visible disabilities than invisible ones and saw this as problematic.

With regards to its autistic employees, it tends to be better at recognising obvious and visible disability. It struggles to cope with less visible, less obvious issues. (PS4)

Navigating the invisibility advocacy dilemma was seen as challenging by some as the ambition to hide the autism or ADHD was argued to impact them in various ways. The dilemma of speaking out or hiding the characteristics of the condition was constructed as ‘*exhausting*’ as many masked their authentic identity at work. For example, PO5 reported feeling a pressure to work in neurotypical ways resulting in *‘masking’*.

And masking. . .it’s exhausting. I genuinely don’t feel that my colleagues have seen me for who I am because I mask so much. . . (PO5)

For this ideological dilemma, one side of the tension was using masking and discretion to remain invisible and not reveal the autism or ADHD to others as a way of staying safe and not drawing attention to their difference. In contrast, participants also raised their concerns about the need for advocacy to promote awareness through education and creating cultural change. The organisational landscape was situated as creating the tension due to stigma and pejorative attitudes which contributed to the dilemma. Thus, the core ideological dilemma with regard to visibility was that to address stigma requires advocacy, but advocacy requires visibility.

## Discussion

Through analysis we demonstrated three ideological dilemmas navigated by participants: (1) tensions in navigating the medical and social identity; (2) tensions between constructing ability and disability; and (3) tensions between remaining invisible and being an advocate. Through close attention to the narratives of the participants we have shown how they identified some of the challenges these created in the real world of policing. Indeed, it was evident that police participants were members of the broader sociocultural society that has medicalisation imbued in its psyche, with its powerful tools for creating mechanisms for the ways in which disabled members are treated and perceived. Yet, these participants were also experts by experience with a lived reality, navigating the fringes of the dilemmas created by the wider discourses and medicalisation of autism and ADHD.

The medicalisation of autism and ADHD has been critically questioned. Arguments posit value in moving away from medicalised language to focus on the cultural, political and social framings of autism and ADHD (Solomon, 2011) and the political movement of neurodiversity has sought to advocate by promoting a discourse of difference. In so doing, there is alignment with the social model of disability, whereby autism and ADHD are seen as socially, culturally and linguistically produced (Orsini, 2012). However, alternatively, some do advocate for the medical explanations and treatments with mobilisation of support and services and they express concern about the removal of autism and ADHD from medical categories (see Tan, 2024). Indeed, psychiatrists in modern medicine typically do recognise the blend of medicine and social understandings seeking to balance the best interests of individuals (Awhansgansi et al., 2023). Yet, medical ideologies are potentially maintained by those who practice psychiatry and psychology by reproducing ideological beliefs through language, that is, through the repertoires of illness, normality and disability.

Tensions continue to exist between the medical model of autism and ADHD positioning ‘deficit’ within the individual, against social model arguments that highlight challenges for neurodivergent people of the ideological neurotypical lens. Such tensions were actively navigated by the participants. Data demonstrated how the insider view of individuals through the lived experience of their occupation against the wider outsider socio-cultural constructions of ‘normality’ and ‘disability’ meant they grappled with positioning the self in policing. This links with broader language arguments in autism where identity first language is favoured (e.g. autistic person, not person with autism; Kenny et al., 2016). In this sense, then, power is relational as the identity of the neurodivergent person is partially dependent on the positioning by others (Wetherell, 1998).

Medicalisation of autism and ADHD is reflected in the broader systemic institutions that perpetuate disability narratives. In such a way, therefore, the participants saw those outsider perspectives as imposing a framing of their identity upon them. It was these tensions between medical constructions of normality and disability, against social model and neurodiversity arguments that were simultaneously accounted for by individuals living and working with autism and ADHD. In managing these tensions, participants considered how the medicalisation of autism and/or ADHD created validation of their condition, and how the formalisation of their neurodivergence brought certain assumptions. They further worked to identify how a neurotypical society created challenges and distanced themselves from the medical self in doing so. Likewise, participants acknowledged that the construction of the condition brought with it inherent assumptions about disability and ability, and the disabled identity was actively resisted. Defining disability and what counts as disabled is complex (Lester and O’Reilly 2021a), and thus in exploring the perspectives of participants, like Ingstad and Whyte (2007), we are not offering concrete definitions of disability, rather we explored how participants constructed and resisted the dilemma of the meanings of ability and disability.

We have demonstrated that the boundaries existing between the medical and the social model, and between medicalisation and neurodiversity, is not as refined in the real lived experiences of individuals. The boundary between the medical and the social model constructs are blended and blurred, and the unique experiences of each person reflects different aspects of both social and medical elements. Participants worked for police organisations and within that employment landscape navigated those boundaries between their self-identity and medical elements of their condition and any potential disabling effects on their work. By focusing on their voices, the ideological ways in which health categories and social situations manifested through employment and exploring the different aspects of identity we illustrated that the lived reality is not simply a reflection of one or the other. Furthermore, the medical ideology is itself is a powerful social construct but nonetheless is still a social construct.

There is little research exploring specific career pathways for neurodivergent people like policing, and even less focusing on the value of examining discourse. Our work provides unique insights into this occupation for this population. Nonetheless, we acknowledge limits of the analysis. In qualitative sampling terms we included a large cohort of 37 voices and had a good representative blend of autism and ADHD, male and female participants, and officers and staff. Nonetheless, consent and inclusion were self-selective as participants expressed an interest via the survey and self-identified as willing to talk. Notably, those who were more reticent to discuss their condition are not heard here and remain hidden. Additionally, while five participants were undiagnosed, epidemiological research illuminates that there are likely many people who are autistic or have ADHD but are unaware (O’Nions et al., 2023). This means some individuals may have not come forward because of uncertainty regarding their status.

In conclusion, we argue it is essential to listen to autistic individuals and those with ADHD about their career experiences as the insider perspective is informative and challenges some of the wider sociocultural power dynamics created through orientations to medicalisation. Furthermore, research needs to explore specific career trajectories as there are different ways in which the outsider view influences an understanding of employment for autistic people or those with ADHD. Our findings could inform new approaches for police organisations in supporting neurodivergent police in ways that help them navigate some of the dilemmas reported. Police organisations could undergo training to improve understanding and attitudes making the environment feel safer to reduce invisibility. Furthermore, awareness of how neurotypical police utilise medicalised language and the potential pejorative constructions this can create are important. The participants’ concerns illuminate a need for police organisations to recognise the strengths these individuals bring to their roles, but also benefits that can be realised in policing because of their characteristics. Perhaps most importantly, the findings illustrate the value in supporting and being mindful of the individuality of each person. This is especially important within an occupation of a uniformed service that relies on uniformity. In recognising uniqueness and ability, even where some characteristics may be disabling, we can move away from binaries and recognise the value of the person even where they need some support in some domains of their lives.
